# Supplementary material for: Yiai Fuzheng decoction inhibits triple-negative breast cancer by remodeling the immune microenvironment
Source: Front Immunol. 2025 Sep 30;16:1615631. doi: 10.3389/fimmu.2025.1615631 (PMC12518410; doi:10.3389/fimmu.2025.1615631)
Supplement: Supplementary file 2 [file DataSheet1.zip › Supplementary File 2/Treatment/C_vs_D_vs_H/06_Pathway_Analysis/Pathway_Analysis/Sig_Pathways_ID/Glycine, serine and threonine metabolism.pdf]

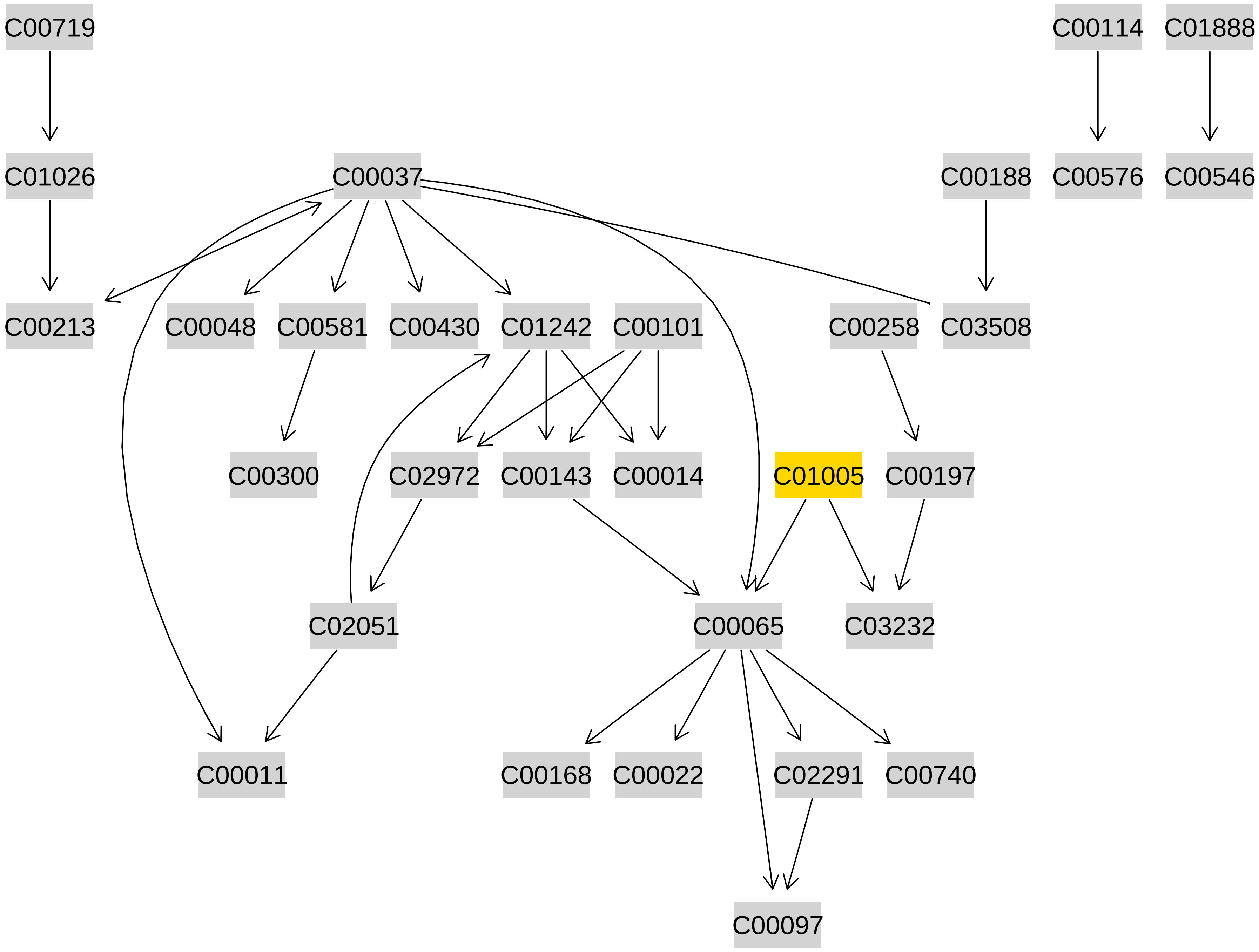
C00719
C00114
C01888
C01026
C00037
C00188
C00576
C00546
C00213
C00048
C00581
C00430
C01242
C00101
C00258
C03508
C00300
C02972
C00143
C00014
C01005
C00197
C02051
C00065
C03232
C00011
C00168
C00022
C02291
C00740
C00097
